# Assessment of thoracic disk herniation by using virtual noncalcium dual-energy CT in comparison with standard grayscale CT

**DOI:** 10.1007/s00330-021-07989-5

**Published:** 2021-06-02

**Authors:** Vitali Koch, Ibrahim Yel, Leon D. Grünewald, Sebastian Beckers, Iris Burck, Lukas Lenga, Simon S. Martin, Christoph Mader, Julian L. Wichmann, Moritz H. Albrecht, Katrin Eichler, Tatjana Gruber-Rouh, Tommaso D’Angelo, Silvio Mazziotti, Giorgio Ascenti, Thomas J. Vogl, Christian Booz

**Affiliations:** 1 Division of Experimental Imaging, Department of Diagnostic and Interventional Radiology, University Hospital Frankfurt, Johann Wolfgang Goethe University, Theodor-Stern-Kai 7, 60590 Frankfurt am Main, Germany; 2 Department of Diagnostic and Interventional Radiology, University Hospital Frankfurt, Johann Wolfgang Goethe University, Frankfurt am Main, Germany; 3 Department of Biomedical Sciences and Morphological and Functional Imaging, “G. Martino” University Hospital Messina, Messina, Italy; 4 Johann Wolfgang Goethe University, Frankfurt am Main, Germany

**Keywords:** Tomography, X-ray computed, Intervertebral disc displacement, Image processing, computer-assisted, Spine

## Abstract

**Objectives:**

To determine the diagnostic accuracy of dual-energy CT (DECT) virtual noncalcium (VNCa) reconstructions for assessing thoracic disk herniation compared to standard grayscale CT.

**Methods:**

In this retrospective study, 87 patients (1131 intervertebral disks; mean age, 66 years; 47 women) who underwent third-generation dual-source DECT and 3.0-T MRI within 3 weeks between November 2016 and April 2020 were included. Five blinded radiologists analyzed standard DECT and color-coded VNCa images after a time interval of 8 weeks for the presence and degree of thoracic disk herniation and spinal nerve root impingement. Consensus reading of independently evaluated MRI series served as the reference standard, assessed by two separate experienced readers. Additionally, image ratings were carried out by using 5-point Likert scales.

**Results:**

MRI revealed a total of 133 herniated thoracic disks. Color-coded VNCa images yielded higher overall sensitivity (624/665 [94%; 95% CI, 0.89–0.96] vs 485/665 [73%; 95% CI, 0.67–0.80]), specificity (4775/4990 [96%; 95% CI, 0.90–0.98] vs 4066/4990 [82%; 95% CI, 0.79–0.84]), and accuracy (5399/5655 [96%; 95% CI, 0.93–0.98] vs 4551/5655 [81%; 95% CI, 0.74–0.86]) for the assessment of thoracic disk herniation compared to standard CT (all *p* < .001). Interrater agreement was excellent for VNCa and fair for standard CT (*ϰ* = 0.82 vs 0.37; *p* < .001). In addition, VNCa imaging achieved higher scores regarding diagnostic confidence, image quality, and noise compared to standard CT (all *p* < .001).

**Conclusions:**

Color-coded VNCa imaging yielded substantially higher diagnostic accuracy and confidence for assessing thoracic disk herniation compared to standard CT.

**Key Points:**

*• Color-coded VNCa reconstructions derived from third-generation dual-source dual-energy CT yielded significantly higher diagnostic accuracy for the assessment of thoracic disk herniation and spinal nerve root impingement compared to standard grayscale CT.*

*• VNCa imaging provided higher diagnostic confidence and image quality at lower noise levels compared to standard grayscale CT.*

*• Color-coded VNCa images may potentially serve as a viable imaging alternative to MRI under circumstances where MRI is unavailable or contraindicated.*

## Introduction

Disk herniation represents a frequent disease, particularly affecting people aged 30 to 50 with an incidence of about 5 to 20 cases per 1000 adults annually [1]. While lumbar disk herniations are most frequently encountered, thoracic disk herniations are rare accounting for less than 1% of all cases [2–5]. However, thoracic disk herniations can result in miscellaneous clinical symptoms including severe myelopathy [4]. Moreover, spinal nerve root impingement can cause severe pain and neurological symptoms. Therefore, immediate detection and localization are crucial for ensuring optimal treatment and prevention of complications.

MRI represents the current standard of reference for visualization of disk herniation because of its ability to provide high contrast between cerebrospinal fluid and intervertebral disks [6, 7]. However, contraindications and limited availability restrict the application of MRI in clinical routine. Therefore, patients with symptoms concerning the thoracic spine initially often undergo X-rays or CT [8]. Compared to MRI, standard grayscale CT offers several advantages including faster examination times and less contraindications but provides only low sensitivity and specificity for assessing herniated disks due to low contrast [9, 10].

Dual-energy CT (DECT) has attracted scientific attention given its unique ability to differentiate various materials by their atomic number [11–15]. In DECT, two different energy spectra are used to measure the attenuation differences, allowing for an improved material characterization and differentiation as well as subtraction of elements with a high atomic number, such as iodine and calcium, resulting in several advantages compared to conventional CT in clinical routine [13, 16]. Numerous studies have shown improved diagnostic accuracy and image quality of DECT for depicting miscellaneous pathologies in the field of cardiovascular, oncological, urological, neurological, and musculoskeletal (MSK) imaging [8, 10, 14–18]. With the advent of third-generation dual-source DECT, different virtual noncalcium (VNCa) reconstruction algorithms have been integrated into postprocessing software, allowing for a time-efficient examination of different clinically relevant issues in MSK imaging. In this context, a novel VNCa postprocessing algorithm established for a color-coded depiction of intervertebral disks has been developed that substantially improves diagnostic accuracy for assessing lumbar and cervical disk herniation compared to standard CT [19, 20]. In contrast to the lumbar spine, detection of thoracic disk herniation is more challenging due to narrower intervertebral spaces and varying volumes and densities. However, no studies have evaluated the diagnostic accuracy of this novel VNCa postprocessing algorithm for the depiction of thoracic disk herniation to date.

We hypothesized that a novel VNCa postprocessing algorithm for color-coded reconstruction may also improve the depiction of thoracic disk herniation and associated spinal nerve root impingement compared to standard grayscale CT. Thus, the purpose of this study was to evaluate the diagnostic accuracy of color-coded DECT VNCa reconstructions for the assessment of thoracic disk herniation compared to standard CT by using MRI as the reference standard.

## Methods

The institutional review board approved this retrospective study. The requirement to obtain written informed consent was waived.

### Study population

**Fig. 1:**
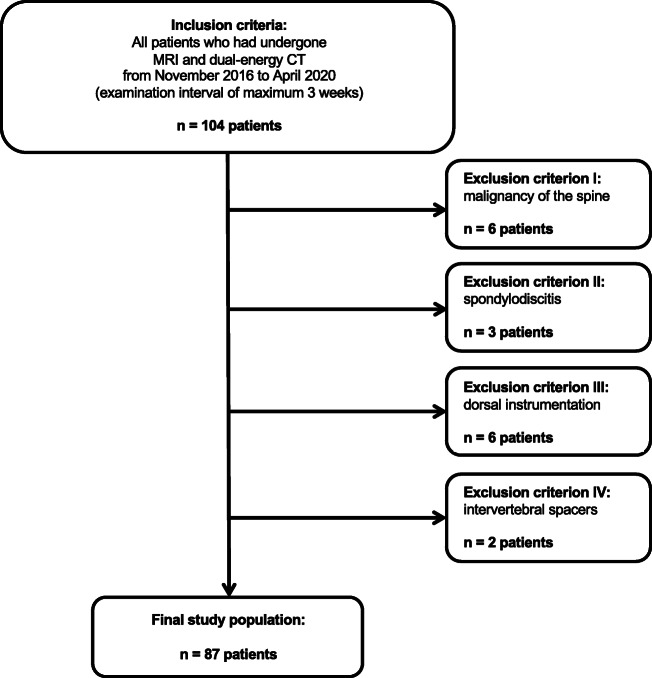
Flowchart of patient inclusion

A total of 104 patients who had undergone routinely performed non-contrast third-generation dual-source DECT and 3.0-T MRI of the thoracic spine due to back pain between November 2016 and April 2020 were candidates for study inclusion. Exclusion criteria were known malignancy of the spine, dorsal instrumentation, spondylodiscitis, and intervertebral spacers. The final data consisted of 87 patients. To ensure comparability and to avoid possible distortion of statistics, only patients with a maximum examination interval of three weeks were included. Figure 1 illustrates the selection process in this study.

### Dual-energy CT scan protocol

All non-contrast CT scans were performed on a third-generation dual-source DECT scanner (Somatom Force; Siemens Healthineers). The system was equipped with two X-ray tubes operating at different voltages (tube A, 90 kVp and 220 mAs; tube B, Sn150 kVp [0.64-mm tin filter] and 138 mAs). CT scans were conducted in craniocaudal scan direction using the following parameters: gantry rotation time, 0.5 s; collimation, 128 × 0.6 mm; pitch, 0.6. Automatic attenuation-based tube current modulation (CARE Dose 4D; Siemens Healthineers) was applied. The mean volume CT dose index was 10.6 mGy ± 3.6 (range, 5.3–15.8 mGy) and the mean dose-length product was 382.5 mGy cm ± 67.1 (range, 157.4–828.1 mGy cm).

### Image reconstruction and postprocessing

From each CT scan, three different image sets were acquired: 90 kVp, Sn150 kVp, and weighted average, which was calculated from the 90 kVp, Sn150 kVp data at a ratio of 0.5:0.5 to simulate a single-energy 120 kVp image [19, 21]. Axial (section thickness of 2 mm and increment of 1 mm) and sagittal (section thickness and increment of 2 mm) images were reconstructed for all three data sets using a medium-soft convolution kernel (Qr40, advanced model-based iterative reconstruction [ADMIRE] level 3/5). Further postprocessing was performed with a commercially available workstation (syngo.via, version VB10B; Siemens Healthineers), based on a three-material decomposition algorithm allowing for differentiation of bone mineral, yellow marrow, and red marrow [10, 22, 23]. The VNCa reconstruction algorithm was applied for the colored analysis of intervertebral disks using dedicated software settings (color lookup table low-energy value, spectrum; color lookup table high-energy value, grayscale; CT preset 1, liver; CT preset 2, bone) according to Booz et al [19]. Colored and grayscale CT series were transferred to a standard picture archiving and communication system (Centricity, version 4.2; GE Healthcare) and hereafter analyzed on a commercially available workstation for radiological image processing (HP Z2 Tower G4 Workstation, Hewlett-Packard GmbH). Reconstruction time for each VNCa image was noted.

### MRI scan protocol

All MRI examinations were non-contrast MRI scans performed on a 3.0-T system (Magnetom PrismaFit; Siemens Healthineers) with a dedicated spine surface coil. The protocol included a standard T1-weighted spin-echo sequence (repetition time ms/echo time ms, 650/10; matrix size, 288 × 384; section thickness, 4 mm), a T2-weighted fast spin-echo sequence (4000/89; matrix size, 358 × 448; section thickness, 4 mm) and a turbo inversion recovery magnitude sequence (3500/39; matrix size, 388 × 384; section thickness, 4 mm). Images were acquired in sagittal orientation except for the T2-weighted sequence which was also performed in the axial plane.

### Image analysis

Image evaluation was performed using the picture archiving and communication system.

To establish the standard of reference, all MRI series were analyzed by two radiologists (T.J.V. and T.G.R., board-certified radiologists with 33 years and 10 years of experience in MSK imaging, respectively) for the presence and degree of thoracic disk herniation based on the classification of the North American Spine Society (NASS) [24] and for the presence (grade 1 vs grades 2–3) of spinal nerve root impingement according to the Pfirrmann nerve root compression grading system [25]. The readers were blinded to DECT data and analyzed MRI series in consensus reading sessions. Diagnostic confidence, image quality, and noise were evaluated individually by using 5-point Likert scales (1, unacceptable; 5, excellent) [26].

After evaluation of MRI series, five readers (L.L., board-certified radiologist with 9 years of experience; I.B., board-certified radiologist with 7 years of experience; J.L.W., radiology resident with 7 years of experience; S.S.M., radiology resident with 5 years of experience; I.Y., radiology resident with 4 years of experience) independently analyzed axial and sagittal DECT series in a randomized blinded fashion. First, standard CT images were assessed for the presence and degree of thoracic disk herniation as well as spinal nerve root impingement on a per-disk basis and on a per-patient basis. After an 8-week interval to prevent recall bias, readers were asked to assess color-coded VNCa reconstructions in the same way without any access to standard grayscale CT data. Image ratings were conducted by using the abovementioned 5-point Likert scales [26].

In general, readers were free to modify window settings of the images and to scroll through the whole stack of CT and MRI series.

### Statistical analysis

Statistical analysis was performed using commercially available software (SPSS Statistics for Windows, version 23.0; IBM, and MedCalc for Windows, version 13; MedCalc). The nonparametric Kolmogorov-Smirnov test was applied to assess the normality of data. Continuous variables were expressed as means ± standard deviation and analyzed with the Wilcoxon test. A *p* < .05 was considered statistically significant.

To compare the diagnostic accuracy of VNCa reconstructions and standard CT series, sensitivity, specificity, as well as accuracy values were calculated on a per-patient basis and on a per-disk basis. Results from clustering of intervertebral disks following the method described by Genders et al [27] were put into a contingency table, comprising each reader and consensus reading. Logistic regression analysis was conducted with a robust variance estimator. Interrater agreement was evaluated using weighted *κ* statistics according to Landis and Koch [28].

## Results

A total of 1131 thoracic intervertebral disks (C7-L1) (median per patient, 13; range, 13) in 87 patients (66 years ± 17; range, 20–94 years), consisting of 40 men (46%; 59 years ± 16; range, 30–87 years) and 47 women (54%; 73 years ± 16; range, 20–94 years), were finally included (Table 1). Seventeen patients were previously excluded owing to implanted osteosynthesis material (6 patients), malignancy (6 patients), spondylodiscitis (3 patients), and intervertebral spacers (2 patients).

**Table 1:** Patient population data (*n* = 87)

Characteristics	Value
Number of overall patients (women; men)	87 (47; 40)
Overall mean age (y) ± SD, range	66 ± 17, 20–94
Overall mean BMI (kg/m^2^) ± SD, range	28 ± 3, 19–38
Mean age of women (y) ± SD, range (mean BMI of women (kg/m^2^) ± SD, range)	73 ± 16, 20–94 (29 ± 5, 19–38)
Mean age of men (y) ± SD, range (mean BMI of men (kg/m^2^) ± SD, range)	59 ± 16, 30–87 (27 ± 3, 19–34)
Number of patients with known thoracic disk herniation	4/87 (5%)
Number of patients with known osteoporosis	12/87 (14%)
Number of patients with known scoliosis	5/87 (6%)

*SD*, standard deviation; *BMI*, body mass index

MRI revealed a total of 133 thoracic herniated disks (12% of all thoracic intervertebral disks; median per patient, 2; range, 1–3) and 25 instances of spinal nerve root impingement. According to the classification of the NASS [24], thoracic disk herniations were classified as 128 protrusions (96%), 4 extrusions (3%), and 1 sequestration (1%). The mean examination interval between DECT and MRI was 7 days (range, 0–16 days). The mean reconstruction time of VNCa images was 2 min (range, 1–3 min).

### Diagnostic accuracy per patient

**Fig. 2:**
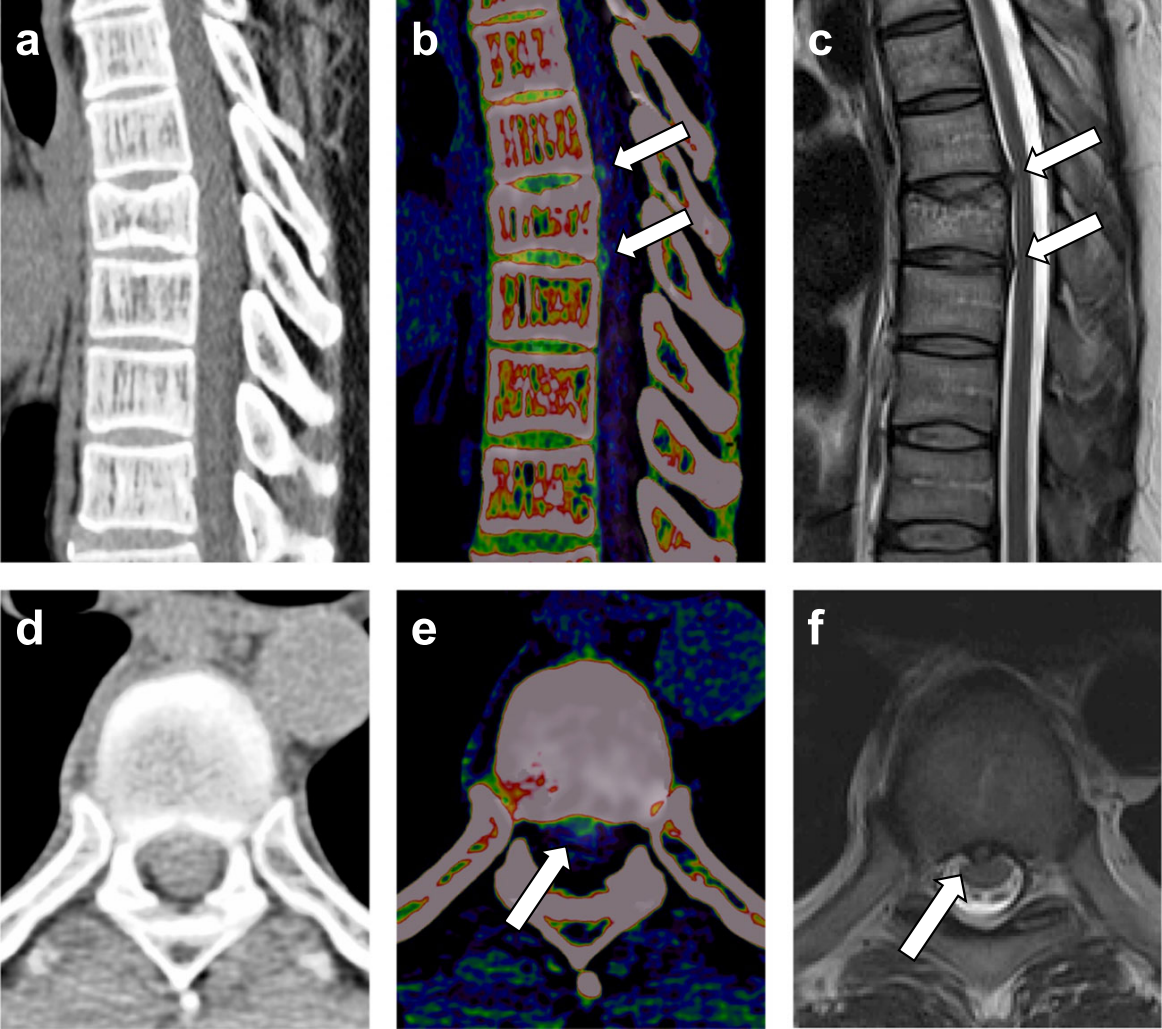
Case of a 59-year-old man who was admitted to the emergency department with severe chest pain radiating to the back for exclusion of posterior myocardial infarction. After ruling out myocardial infarction, spine dual-energy CT imaging was performed to exclude fractures due to persistent symptoms. Grayscale CT series showed an old cover plate impression of Th9 on a sagittal plane. Due to low contrast between thoracic disks and cerebrospinal fluid on the sagittal and transversal plane (**a**, **d**), sagittal and transversal color-coded virtual noncalcium (VNCa) images (**b**, **e**) were reconstructed for assessing thoracic disk herniations. Sagittal VNCa reconstructions depicted median thoracic disk protrusions at level Th8/9 and Th9/10 (*arrows*), which were also seen on transversal VNCa images (thoracic disk protrusion at level Th8/9 is illustrated). Both thoracic disk protrusions were not clearly visible on the sagittal and transversal grayscale CT series. MRI confirmed the diagnosis of median thoracic disk protrusions at level Th8/9 and Th9/10 on sagittal and transversal T2-weighted series (*arrows*) (**c**, **f**)

The patient-based analysis demonstrated higher overall sensitivity (118/123 [96%; 95% CI, 0.92–0.99] vs 92/123 [75%; 95% CI, 0.60–0.92]), specificity (290/312 [93%; 95% CI, 0.87–0.97] vs 270/312 [87%; 95% CI, 0.79–0.97]), and accuracy (408/435 [94%; 95% CI, 0.89–0.98] vs 362/435 [83%; 95% CI, 0.75–0.92]) of color-coded VNCa reconstructions vs standard CT for the assessment of thoracic disk protrusion (all comparisons, *p* < .001). Figure 2 shows an example case illustrating the potential to improve the detection of thoracic disk herniation by application of color-coded VNCa images.

Regarding the depiction of spinal nerve root impingement, VNCa images yielded higher overall sensitivity (87/95 [92%; 95% CI, 0.85–0.96] vs 60/95 [63%; 95% CI, 0.48–0.81]), specificity (325/340 [96%; 95% CI, 0.94–0.98] vs 297/340 [87%; 95% CI, 0.78–0.96]), and accuracy (412/435 [95%; 95% CI, 0.91–0.98] vs 357/435 [82%; 95% CI, 0.74–0.91]) as compared to standard CT (all comparisons, *p* < .001). Table 2 summarizes the patient-based diagnostic accuracy results for classification of thoracic disk herniation as well as for assessing spinal nerve root impingement.

**Table 2:** Patient-based diagnostic accuracy of standard grayscale CT and color-coded VNCa reconstructions for the classification of thoracic disk herniation. The analysis showed higher overall sensitivity, specificity, and accuracy of color-coded VNCa reconstructions compared with standard grayscale CT

Patient-based	Sensitivity	Specificity	Accuracy
Thoracic disk protrusion			
Standard CT	75% (92/123)	87% (270/312)	**83%** (362/435)
	[0.60–0.92]	[0.79–0.97]	[0.75–0.92]
VNCa	96% (118/123)	93% (290/312)	**94%** (408/435)
	[0.92–0.99]	[0.87–0.97]	[0.89–0.98]
Thoracic disk extrusion			
Standard CT	73% (11/15)	96% (403/420)	**95%** (414/435)
	[0.60–0.88]	[0.93–0.98]	[0.92–0.98]
VNCa	100% (15/15)	99% (417/420)	**99%** (432/435)
		[0.98–1.00]	[0.97–1.00]
Thoracic disk sequestration			
Standard CT	80% (4/5)	98% (423/430)	**98%** (427/435)
	[0.69–0.90]	[0.97–0.99]	[0.96–0.99]
VNCa	100% (5/5)	99% (426/430)	**99%** (431/435)
		[0.98–1.00]	[0.98–1.00]
Spinal nerve root impingement			
Standard CT	63% (60/95)	87% (297/340)	**82%** (357/435)
	[0.48–0.81]	[0.78–0.96]	[0.74–0.91]
VNCa	92% (87/95)	96% (325/340)	**95%** (412/435)
	[0.85–0.96]	[0.94–0.98]	[0.91–0.98]

*VNCa*, virtual noncalcium. Data in parentheses are numerators and values in square brackets are 95% confidence intervals. Accuracy percentages are displayed in bold

Interrater agreement was excellent for VNCa reconstructions and moderate for standard CT images regarding the depiction of thoracic disk protrusion (*ϰ* = 0.85 [95% CI, 0.80–0.91] vs *ϰ* = 0.60 [95% CI, 0.51–0.68]), excellent and substantial for thoracic disk extrusion (*ϰ* = 0.91 [95% CI, 0.80–1.00] vs *ϰ* = 0.82 [95% CI, 0.67–0.97]), excellent and excellent for thoracic disk sequestration (*ϰ* = 1.00 [95% CI, 1.00–1.00] vs *ϰ* = 1.00 [95% CI, 1.00–1.00]), and excellent and moderate for spinal nerve root impingement (*ϰ* = 0.85 [95% CI, 0.79–0.91] vs *ϰ* = 0.49 [95% CI, 0.39–0.59]) (all comparisons, *p* < .001).

### Diagnostic accuracy per intervertebral disk

Analysis per intervertebral disk revealed higher overall sensitivity (624/665 [94%; 95% CI, 0.89–0.96] vs 485/665 [73%; 95% CI, 0.67–0.80]), specificity (4775/4990 [96%; 95% CI, 0.90–0.98] vs 4066/4990 [82%; 95% CI, 0.79–0.84]), and accuracy (5399/5655 [96%; 95% CI, 0.93–0.98] vs 4551/5655 [81%; 95% CI, 0.74–0.86]) of color-coded VNCa reconstructions for the depiction of thoracic disk herniation in comparison to standard CT images taking clustering into account (all comparisons, *p* < .001) (Table 4). Interrater agreement was excellent for VNCa images (*ϰ* = 0.82 [95% CI, 0.78–0.83]) and fair for standard CT (*ϰ* = 0.37 [95% CI, 0.34–0.40]) (*p* < .001).

In terms of thoracic disk protrusion, statistical analysis demonstrated higher overall sensitivity (622/660 [94%; 95% CI, 0.90–0.97] vs 487/660 [74%; 95% CI, 0.67–0.81]), specificity (4794/4995 [96%; 95% CI, 0.94–0.99] vs 4071/4995 [82%; 95% CI, 0.79–0.84]), and accuracy (5416/5655 [96%; 95% CI, 0.93–0.98] vs 4558/5655 [81%; 95% CI, 0.75–0.87]) of VNCa reconstructions (all comparisons, *p* < .001) (Table 3). Interrater agreement was excellent for VNCa images (*ϰ* = 0.82 [95% CI, 0.79–0.84]) and fair for standard CT (*ϰ* = 0.37 [95% CI, 0.34–0.40]) (*p* < .001).

**Table 3:** Disk-based diagnostic accuracy of standard grayscale CT and color-coded VNCa reconstructions for the classification of thoracic disk herniation. VNCa reconstructions yielded higher diagnostic accuracy for the correct classification of thoracic disk herniation and detection of spinal nerve root impingement as compared to standard grayscale CT

Disk-based	Sensitivity	Specificity	Accuracy
Thoracic disk protrusion			
Standard CT	74% (487/660)	82% (4071/4995)	**81%** (4558/5655)
	[0.67–0.81]	[0.79–0.84]	[0.75–0.87]
VNCa	94% (622/660)	96% (4794/4995)	**96%** (5416/5655)
	[0.90–0.97]	[0.94–0.99]	[0.93–0.98]
Thoracic disk extrusion			
Standard CT	45% (9/20)	95% (5370/5635)	**95%** (5379/5655)
	[0.23–0.74]	[0.92–0.98]	[0.93–0.98]
VNCa	95% (19/20)	97% (5487/5635)	**97%** (5506/5655)
	[0.90–0.98]	[0.95–1.0]	[0.95–0.99]
Thoracic disk sequestration			
Standard CT	60% (3/5)	96% (5445/5650)	**96%** (5448/5655)
	[0.40–0.80]	[0.94–0.99]	[0.93–0.99]
VNCa	100% (5/5)	99% (5608/5650)	**99%** (5613/5655)
		[0.97–1.0]	[0.98–1.0]
Spinal nerve root impingement			
Standard C	61% (85/140)	91% (4995/5515)	**90%** (5080/5655)
	[0.49–0.74]	[0.86–0.95]	[0.85–0.93]
VNCa	96% (135/140)	98% (5420/5515)	**98%** (5555/5655)
	[0.93–0.98]	[0.95–1.0]	[0.96–1.0]

*VNCa*, virtual noncalcium. Data in parentheses are numerators and values in square brackets are 95% confidence intervals. Accuracy percentages are displayed in bold

**Fig. 3:**
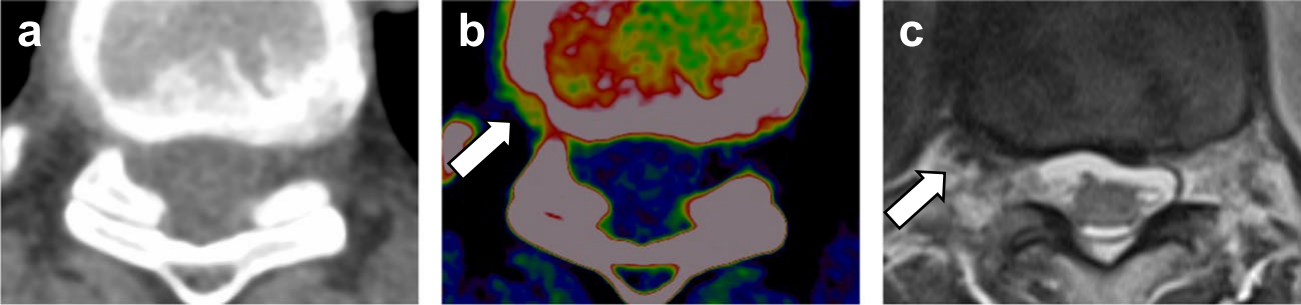
Case of a 67-year-old woman suffering from right-sided back pain who underwent spine dual-energy CT imaging. During analysis of transversal color-coded virtual noncalcium images (**b**), right-sided impingement of Th8 (contact, grade 1 according to the Pfirrmann nerve root compression grading system) due to disk bulging was detected by all readers in this study (*arrow*), which was initially missed on transversal grayscale CT series (**a**) by 3/5 readers. Additionally performed axial T2-weighted MRI series confirmed right-sided grade 1 spinal nerve root impingement (*arrow*) (**c**)

Regarding the assessment of spinal nerve root impingement, VNCa reconstructions yielded higher overall sensitivity (135/140 [96%; 95% CI, 0.93–0.98] vs 85/140 [61%; 95% CI, 0.49–0.74]), specificity (5420/5515 [98%; 95% CI, 0.95–1.0] vs 4995/5515 [91%; 95% CI, 0.86–0.95]), and accuracy (5555/5655 [98%; 95% CI, 0.96–1.0] vs 5080/5655 [90%; 95% CI, 0.85–0.93]) compared to standard CT taking clustering into account (all comparisons, *p* < .001). Interrater agreement was substantial for VNCa images (*ϰ* = 0.72 [95% CI, 0.67–0.77]) and poor for standard CT (*ϰ* = 0.20 [95% CI, 0.16–0.24]). An example case showing facilitated assessment of spinal nerve root impingement by using VNCa reconstructions is illustrated in Fig. 3.

When comparing the results of each CT reader, the least experienced reader showed the most distinct improvement by using color-coded VNCa reconstructions for assessing thoracic disk herniation with higher overall sensitivity (121/133 [91%; 95% CI, 0.85–0.95] vs 89/133 [67%; 95% CI, 0.60–0.84]), specificity (939/998 [94%; 95% CI, 0.90–0.97] vs 787/998 [79%; 95% CI, 0.70–0.86]), and accuracy (1060/1131 [94%; 95% CI, 0.90–0.97] vs 876/1131 [78%; 95% CI, 0.71–0.84]) compared to standard CT (all comparisons, *p* < .001). The most experienced reader was able to achieve excellent diagnostic performance in assessing thoracic disk herniation with an overall sensitivity of 96% (128/133 [95% CI, 0.92–0.99]), specificity of 95% (951/998 [95% CI, 0.88–0.98]), and accuracy of 95% (1079/1131 [95% CI, 0.91–0.97]). Likewise, the other three readers achieved significantly higher sensitivity, specificity, and accuracy (all comparisons, *p* < .001) levels for the detection of thoracic disk herniation and spinal nerve root impingement compared to standard CT (Table 4).

**Table 4:** Diagnostic accuracy of each reader for the detection of thoracic disk herniation per intervertebral disk. Diagnostic accuracy of all readers improved significantly by using VNCa reconstructions. Particularly, the most experienced reader (reader 3, board-certified radiologist with 9 years of experience) yielded excellent diagnostic accuracy assessing thoracic disk herniation using color-coded VNCa reconstructions. Experience level in MSK imaging: reader 1, 5 years; reader 2, 7 years; reader 3, 9 years; reader 4, 4 years; and reader 5, 7 years

		Sensitivity	Specificity	Accuracy
Average	Standard CT	73% (485/665)	82% (4066/4990)	**81%** (4551/5655)
		[0.67–0.80]	[0.79–0.84]	[0.74–0.86]
	VNCa	94% (624/665)	96% (4775/4990)	**96%** (5399/5655)
		[0.89–0.96]	[0.90–0.98]	[0.93–0.98]
Reader 1	Standard CT	72% (96/133)	83% (828/998)	**82%** (924/1131)
		[0.62–0.89]	[0.77–0.89]	[0.77–0.87]
	VNCa	93% (123/133)	96% (953/998)	**95%** (1076/1131)
		[0.88–0.96]	[0.93–0.98]	[0.90–0.98]
Reader 2	Standard CT	71% (94/133)	84% (842/998)	**83%** (936/1131)
		[0.57–0.87]	[0.79–0.91]	[0.78–0.89]
	VNCa	95% (126/133)	97% (969/998)	**97%** (1095/1131)
		[0.89–0.98]	[0.93–0.99]	[0.94–0.99]
Reader 3	Standard CT	75% (100/133)	81% (803/998)	**80%** (903/1131)
		[0.66–0.85]	[0.75–0.86]	[0.75–0.85]
	VNCa	96% (128/133)	95% (951/998)	**95%** (1079/1131)
		[0.92–0.99]	[0.88–0.98]	[0.91–0.97]
Reader 4	Standard CT	67% (89/133)	79% (787/998)	**78%** (876/1131)
		[0.60–0.84]	[0.70–0.86]	[0.71–0.84]
	VNCa	91% (121/133)	94% (939/998)	**94%** (1060/1131)
		[0.85–0.95]	[0.90–0.97]	[0.90–0.97]
Reader 5	Standard CT	80% (106/133)	81% (806/998)	**81%** (912/1131)
		[0.72–0.90]	[0.72–0.89]	[0.74–0.88]
	VNCa	95% (126/133)	97% (963/998)	**96%** (1089/1131)
		[0.92–0.97]	[0.94–0.99]	[0.92–0.99]

*VNCa*, virtual noncalcium. Data in parentheses are numerators and values in square brackets are 95% confidence intervals. Accuracy percentages are displayed in bold

### Image ratings

**Fig. 4:**
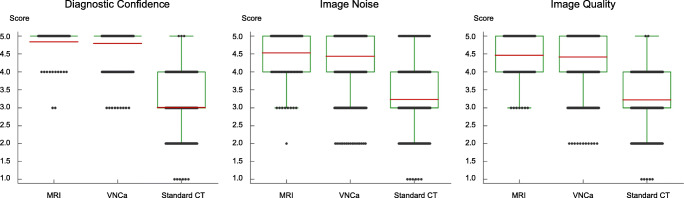
Box and dot plots illustrate subjective image rating results regarding the diagnostic reader confidence, image noise, and quality of MRI, standard grayscale CT, and color-coded virtual noncalcium (VNCa) series. Mean scores are shown as horizontal red lines and dots represent the distribution of scores. Ratings for color-coded VNCa reconstructions were significantly higher concerning all three categories compared to standard grayscale CT images (all *p* < .001). Ratings for VNCa reconstructions and MRI series differed not significantly for diagnostic confidence (*p* = .38), image noise (*p*= .36), and image quality (*p* = .61)

Readers showed high diagnostic confidence using MRI series and color-coded VNCa reconstructions for assessing thoracic disk herniation without significant differences (4.84 ± 0.43 and 4.79 ± 0.45, respectively) (*p =* .38) (Fig. 4). Concerning standard CT images, ratings showed lower confidence (3.01 ± 0.76) as compared to VNCa reconstructions (*p* < .001). In this context, interrater agreement was excellent for VNCa images (*ϰ* = 0.84 [95% CI, 0.77–0.91]) and substantial for standard CT (*ϰ* = 0.80 [95% CI, 0.75–0.85]).

Image quality was rated with mean scores of 4.46 ± 0.64 for MRI and 4.41 ± 0.80 for VNCa reconstructions, showing no significant difference between both modalities (*p* = .61). In contrast, image quality ratings for standard CT were lower in comparison to VNCa reconstructions with a mean score of 3.22 ± 0.74 (*p* < .001). Interrater agreement was excellent for VNCa (*ϰ* = 0.88 [95% CI, 0.83–0.92]) and standard CT (*ϰ* = 0.82 [95% CI, 0.75–0.89]).

The image noise varied not significantly (*p =* .36) between MRI series (4.53 ± 0.71) and VNCa reconstructions (4.44 ± 0.91). However, noise levels were rated as being more present on standard CT images (3.23 ± 0.83) when compared with VNCa images (*p* < .001). Interrater agreement was excellent for both, VNCa images (*ϰ* = 0.86 [95% CI, 0.80–0.91]) and standard CT (*ϰ* = 0.83 [95% CI, 0.79–0.87]).

## Discussion

This study demonstrated first that color-coded VNCa reconstructions derived from third-generation dual-source DECT improve the depiction of thoracic disk herniation and spinal nerve root impingement with higher diagnostic accuracy compared to standard grayscale CT. Furthermore, VNCa imaging provided greater diagnostic confidence and higher image quality at lower noise levels. Concomitantly, VNCa reconstructions yielded higher levels of interrater agreement in comparison to standard CT, suggesting greater diagnostic reliability of this recently developed DECT postprocessing algorithm.

While only moderate diagnostic accuracy has been reported for the depiction of disk herniation at different levels using standard CT [9, 26], the applied VNCa algorithm optimized for the visualization of intervertebral disks has been recently evaluated for assessing lumbar and cervical disk herniation and has revealed substantially higher diagnostic accuracy and confidence compared to standard CT [19, 20]. Considering the obviously different anatomy of the thoracic spine including varying intervertebral spaces and different volumes and densities compared to the lumbar and cervical spine, it is questionable whether VNCa data regarding cervical and lumbar disk herniation assessment are transferable to the thoracic spine. Compared to the lumbar spine, intervertebral spaces of the thoracic spine are narrower with different consistencies of spinal fluid and disks [29]. In contrast to the cervical spine being the most mobile part of the spine, the thoracic spine represents the most stable and least flexible part. Thoracic vertebral foramina are comparatively small and intervertebral disks are thin relative to the larger and heart-shaped vertebral bodies [29]. In addition, the thoracic spine provides attachment sites for the rib cage resulting in different forces. While lordosis is commonly present in the cervical and lumbar spine, a kyphosis is the main finding in the thoracic spine. Finally, deformities such as scoliosis with consecutive degenerative changes of the thoracic spinal column frequently hamper the correct assessment of disk herniation and nerve root impingement [26]. Therefore, our aim was to investigate if color-coded VNCa reconstructions also improve diagnostic accuracy for the assessment of thoracic disk herniation compared to standard grayscale CT. In this context, our study results demonstrate high accuracy of color-coded VNCa images in depicting herniated disks also in the thoracic spine. The substantially different architecture of the thoracic, lumbar, and cervical regions did not impact the diagnostic capability of the proposed approach for reconstruction of VNCa images to depict vertebral disk pathologies. Diagnostic accuracy of colored VNCa reconstructions for depicting disk herniation in comparison with standard grayscale CT was similar concerning lumbar (92% vs 82%, *p* < .001), cervical (95% vs 77%, *p* < .001), and thoracic spine (96% vs 81%, *p* < .001) [19, 20]. In contrast with previous studies, the diagnostic accuracy for depicting intervertebral disks was unaffected by degenerative spinal changes, such as spondylophytes, or narrowed intervertebral spaces [19, 26]. This study emphasizes the potential of DECT to serve as a viable spine imaging alternative to MRI in cases of MRI contraindications or limited availability.

Comparing VNCa reconstructions and standard CT series regarding the correct classification of thoracic disk herniation, readers experienced the greatest benefit using VNCa images in case of extrusions and sequestrations (both sensitivity and specificity, ≥ 99%). Moreover, substantially improved diagnostic accuracy was achieved using VNCa imaging for the depiction of small protrusions, especially due to its ability to provide high contrast between disk and cerebrospinal fluid. In general, the most inexperienced reader in our study showed the most significant improvement by using VNCa reconstructions with substantially increased sensitivity (91% vs 67%) as compared to standard CT. This result indicates that the additionally reconstructed VNCa images may particularly improve the diagnostic accuracy of radiology residents for assessing thoracic disk herniation. Another important aspect is the superior readers’ diagnostic confidence when using VNCa reconstructions, which represents an additional essential criterion for making a reliable diagnosis. A short reconstruction time of 2 min on average demonstrates time efficiency and applicability in clinical routine; therefore, we strongly recommend reconstruction of colored VNCa images in case of DECT-based thoracic spine imaging and suspected disk herniation.

In the recent past, DECT has become a widespread innovative imaging modality covering many fields of indications in oncological, cardiovascular, and MSK imaging which is also increasingly installed in middle-sized and small hospitals. The current DECT imaging is performable with almost every new CT system distributed by all major manufacturers. In this context, different methods such as dual-source, rapid kilovoltage switching, and dual-layer technologies are applied to enable DECT imaging. From a technical perspective, image acquisition using DECT with predefined presets and protocols does not cause any substantial time delay or increased radiation dose compared to conventional CT. Third-generation dual-source DECT allows for improved material quantification and differentiation due to its usage of different keV levels and three-material decomposition algorithms [13, 30]. Regarding MSK imaging, studies have shown high diagnostic accuracy of DECT-based techniques for the depiction of bone marrow edema, gout crystals, and injuries of ligaments and tendons in the last years [10, 12–15, 31, 32]. Given its higher spatial resolution in comparison to MRI, the DECT technique and its postprocessing algorithms such as VNCa imaging may provide a more detailed and comprehensive visualization of spinal structures such as osseous as well as soft tissue structures including ligaments and intervertebral disks. In case of trauma, DECT-based thoracic spine imaging may allow for a combined analysis of fractures and associated bone marrow edema as well as disk herniations and ligamentous injuries [9, 33]. Consequently, DECT may serve as a viable imaging alternative to MRI in cases of MRI contraindications. In addition, DECT could potentially enable time and cost savings as well as improved patient comfort without the need for repeated transport and positioning in case of limited MRI availability (e.g., night shifts). Considering the continuously increasing number of CT scans performed in DE mode due to manifold postprocessing applications, the option for opportunistic assessment of thoracic disk herniation represents another crucial advantage over standard CT.

This study had certain limitations. First, our investigations were conducted as a retrospective single-center study which has led to a limited number of patients. Second, examinations were performed in a defined order with analysis of standard CT prior to the assessment of color-coded VNCa reconstructions, potentially causing statistical distortion due to recall bias. Third, due to the lack of a generally accepted classification system for grading thoracic disk herniation to date, we applied the NASS classification system despite its focus on lumbar disk herniation. Fourth, only non-contrast CT scans were evaluated; the influence of contrast material remains unknown. Fifth, our study findings are only restricted to a vendor-specific CT system and may therefore not be transferable to DECT technologies merchandised by other manufacturers. Finally, MRI may have overestimated the degree of disk herniation and spinal nerve root impingement [34, 35]. It was chosen as a reference standard instead of CT myelography for comparison of grayscale and VNCa images due to its high diagnostic value in assessing disk herniation and spinal nerve root impingement as well as in order to provide comparability with previous studies. Standard grayscale CT examinations of the thoracic spine are frequently performed in addition to MRI for further evaluation of fractures and osseous degenerations. By contrast, CT myelography is not regularly performed at our institute if MRI scanning is available as it represents an invasive alternative imaging modality which potentially causes severe complications.

In conclusion, this study demonstrated that color-coded dual-source DECT derived VNCa imaging improves diagnostic accuracy and confidence for assessing thoracic disk herniation and associated spinal nerve root impingement compared with standard grayscale CT using MRI as a standard of reference. Therefore, VNCa reconstructions may serve as a viable imaging alternative to MRI in case of contraindications or MRI unavailability and the possibility to perform DECT.

## Abbreviations

BMI: Body mass index
CI: Confidence interval
DECT: Dual-energy computed tomography
MSK: Musculoskeletal
NPV: Negative predictive value
PPV: Positive predictive value
VNCa: Virtual noncalcium

## Declarations

### Guarantor

The scientific guarantor of this publication is Prof. Dr. Vogl.

### Statistics and biometry

No complex statistical methods were necessary for this paper.

### Methodology

• Retrospective

• Diagnostic or prognostic study

• Performed at one institution
